# Data Enhancement via Low-Rank Matrix Reconstruction in Pulsed Thermography for Carbon-Fibre-Reinforced Polymers

**DOI:** 10.3390/s21217185

**Published:** 2021-10-29

**Authors:** Samira Ebrahimi, Julien R. Fleuret, Matthieu Klein, Louis-Daniel Théroux, Clemente Ibarra-Castanedo, Xavier P. V. Maldague

**Affiliations:** 1 Computer Vision and Systems Laboratory (CVSL), Department of Electrical and Computer Engineering, Laval University, Quebec, QC G1V 0A6, Canada; julien.fleuret.1@ulaval.ca (J.R.F.); clemente.ibarra-castanedo@gel.ulaval.ca (C.I.-C.); Xavier.Maldague@gel.ulaval.ca (X.P.V.M.); 2 Visiooimage Inc. Infrared Thermography Testing Systems, Quebec, QC G1W 1A8, Canada; matthieu.klein@visiooimage.com; 3 Centre Technologique et Aérospatial (CTA), Saint-Hubert, QC 3Y 8Y9, Canada; louis-daniel.theroux@cegepmontpetit.ca

**Keywords:** Robust PCA, RPCA, PCP, IALM, noise reduction, pulsed thermography, CFRP

## Abstract

Pulsed thermography is a commonly used non-destructive testing method and is increasingly studied for the assessment of advanced materials such as carbon fibre-reinforced polymer (CFRP). Different processing approaches are proposed to detect and characterize anomalies that may be generated in structures during the manufacturing cycle or service period. In this study, matrix decomposition using Robust PCA via Inexact-ALM is investigated as a pre- and post-processing approach in combination with state-of-the-art approaches (i.e., PCT, PPT and PLST) on pulsed thermography thermal data. An academic sample with several artificial defects of different types, i.e., flat-bottom-holes (FBH), pull-outs (PO) and Teflon inserts (TEF), was employed to assess and compare defect detection and segmentation capabilities of different processing approaches. For this purpose, the contrast-to-noise ratio (CNR) and similarity coefficient were used as quantitative metrics. The results show a clear improvement in CNR when Robust PCA is applied as a pre-processing technique, CNR values for FBH, PO and TEF improve up to 164%, 237% and 80%, respectively, when compared to principal component thermography (PCT), whilst the CNR improvement with respect to pulsed phase thermography (PPT) was 77%, 101% and 289%, respectively. In the case of partial least squares thermography, Robust PCA results improved not only only when used as a pre-processing technique but also when used as a post-processing technique; however, this improvement is higher for FBHs and POs after pre-processing. Pre-processing increases CNR scores for FBHs and POs with a ratio from 0.43% to 115.88% and from 13.48% to 216.63%, respectively. Similarly, post-processing enhances the FBHs and POs results with a ratio between 9.62% and 296.9% and 16.98% to 92.6%, respectively. A low-rank matrix computed from Robust PCA as a pre-processing technique on raw data before using PCT and PPT can enhance the results of 67% of the defects. Using low-rank matrix decomposition from Robust PCA as a pre- and post-processing technique outperforms PLST results of 69% and 67% of the defects. These results clearly indicate that pre-processing pulsed thermography data by Robust PCA can elevate the defect detectability of advanced processing techniques, such as PCT, PPT and PLST, while post-processing using the same methods, in some cases, can deteriorate the results.

## 1. Introduction

Due to the unique features of Carbon-fibre-reinforced polymers (CFRP)—low-density and high-performance physico-chemical properties—the interest in using these lighter products and thus replacing the conventional materials (Steel, aluminum, etc.) has increased. The increasing demand for CFRP structures in the aerospace industry is leading to the development of enhanced more eco-efficient manufacturing [1]. Although composite materials are sensitive to impact damage during a lifetime (manufacturing, operations, or maintenance) [2], they are less prone to corrosion and cracks than other materials. Due to the different types of defects during the manufacturing process or the service life of the components, it is important to monitor their efficiency and functionality non-invasively. Among non-destructive testing techniques, infrared thermography, which involves mapping the surface temperatures, can characterize the surface and sub-surface anomalies. Pulsed thermography (PT) is a no-contact and full-field Infrared Non-Destructive Testing (IRNDT) approach based on thermal heat transfer analysis during the cooling period; after the thermal impulse, an incident to the sample’s surface becomes a thermal wave due to conduction and propagates through the material. The temperature decay is recorded by the infrared camera during the cooling period. Subject to the presence of discontinuity, depending on its material and thermal properties and depth, defects will be revealed at different times. The deeper defects appear later with lower thermal contrast. In order to obtain quantitative information from thermal data, several approaches have been proposed. Manipulating thermal data makes active thermography an attractive and powerful approach for industrial control and maintenance purposes.

Moreover, effective pre-processing or post-processing can provide favorable conditions to enhance defect information extraction. Most of the pre-processing for thermal data is limited to removing the first few frames from the beginning of the sequences, cropping the image, and selecting the region of interest (ROI). Fleuret et al. [3], in their study, proved that using LatLRR (Latent Low-Rank Representation) as a post-processing tool on the best image of state-of-the-art methods provides significant improvement in detection. Khodayar et al. [4] have used the thermographic signal reconstruction (TSR) [5] approach for pre-processing to reduce the noise. They stated that principal component thermography (PCT) [6] after the noise reduction could enhance the results. Wang et al. [7] used sequence differential pre-processing, which was combined with cold image subtraction (CIS) [8], to provide better thermal data for post-processing approaches in laser infrared thermography. They evaluated the quality of the image after the combination of pre-processing with pulsed phase thermography (PPT) [9] or PCT and found that pre-processing improved some results. Ebrahimi et al. [10] showed that the low-rank matrix computed by RPCA-PCP via Inexact ALM when used with PT data does not provide optimal results; nonetheless, this method has not been investigated as a pre-processing method nor as a post-processing method. Several state-of-the-art IRNDT methods, i.e., PPT, PCT and Partial Least Square Thermography (PLST) [11,12], have been chosen to evaluate the approaches. We chose these methods due to the large number of studies that use them.

In the remainder of this paper, we review the most recent works involving RPCA and thermography. Then, we detail the many aspects of our investigations in Section 3. Section 4 demonstrates the obtained results, which we analyze and discuss in Section 5. Finally, Section 6 concludes this study.

## 2. Literature Review

The presence of excessive noise in raw thermal data always urges researchers to develop new IRNDT processing approaches. Although limited research work has been done on the improvement of PCA methods to deal with corrupted data, RPCA has been the most promising approach in recent years. RPCA is widely used in separating dynamic variations from the static feature of interest, such as video surveillance data analysis to extract foreground and background [13]. Infrared dim small target detection has been a hot and difficult research topic in infrared search and tracking systems. Later, Fan et al. [14] introduced a novel detection algorithm based on RPCA to solve the difficulty of small target detection.

Substantial progress has been made in moving object detection, for which RPCA has been demonstrated to be very effective. The RPCA has been used in infrared moving target tracking [15] and hyper-spectral image processing for anomaly detection [16]. Moreover, RPCA has been used for pre-processing in the machine learning method proposed by Zhu et al. [17]. They utilized RPCA to detect regions of interest (ROIs) in a novel classification model based on the CNN model in eddy current testing (ECT), and the percentage of defects correctly identified have increased to almost 100%. Draganov et al. [18] used several decomposition techniques, such as RPCA with Go implementation (GoDec), to estimate the wild animal population using videos captured by thermographic cameras. They reported promising results in terms of accuracy and execution times. Later, they carried out a comparative analysis of the performance of several tensor decomposition algorithms, including high-order robust principal component analysis solved by the Singleton model (HoRPCA-S) [19]. They reported that among the selected methods, HoRPCA-S has a lower detection rate but high precision. Furthermore, Liang et al. [20] have demonstrated the feasibility of sparse tensor decomposition theory on an ECPT data sequence, and they concluded that Tensor RPCA (TRPCA) can extract defects with high accuracy. The same year, Li et al. [21] introduced the weighted contraction IALM (WIALM) algorithm based on low-rank matrix recovery for online applications. It has been used for tire inspection on radiographic images captured by tire X-ray inspection machines. They improved the efficiency of the algorithm by optimizing the incremental multiplier parameter. Wu et al. [22] proposed a novel hierarchical low-rank and sparse tensor decomposition method to detect anomalies in the induction thermography stream. This approach can suppress the interference of a strong background and sharpens the visual features of defects. Furthermore, it overcame the over- and under-sparseness problem suffered by similar state-of-the-art methods. Surface defect detection is important for product quality control. A visual detection method was based on low-rank and sparse matrices extracted from the RPCA approach for surface defect detection of the wind turbine blade [23]. This method in terms of robustness and accuracy outperformed several state-of-the-art methods. Recently, Wang et al. [24] proposed a methodology based on RPCA that can separate anomalies in a sparse matrix from a low-rank background for photovoltaic systems using thermography imaging. They successfully overcame the difficulties arising from real data and built an automatic online monitoring system for anomaly detection. Ebrahimi et al. [10] proposed the orthogonal inexact augmented lagrange multiplier (OIALM). This study demonstrates its efficiency for defect enhancement capabilities over mixed and various types of defects typically addressed in IRT in composite materials. In addition, Kaur et al. [25] conducted a comparative study between PCA and RPCA to evaluate their effectiveness in defect detection. They demonstrated that although PCA proved to be better in detection capability, the sparse matrix provides better detectability than the data reconstructed from the low-rank matrix. In the medical field, for 3D segmentation of lungs, Sun et al. [26] achieved good segmentation results for lungs with juxta-pleural tumours by the active shape model (ASM) based on RPCA.

Many research works have reported the applicability of IRNDT approaches, including PCT, PPT and PLST. The first implementation of the PCT was introduced by Rajic [27] for defect detection in composite materials. Lara et al. expressed that optical effects, such as heating non-uniformities, surface reflection and emissivity variations, appear on the first component, and the thermal effect will be retrieved on one of the secondary components [28]. Furthermore, the PCA is a linear decomposition function that is sensitive to over-illumination and non-uniform heating more than other types of noise. In our previous research, we proved that Robust PCT [10] can improve the detectability of deeper defects in composites. Moreover, the PLST is sensitive to gradient. Having an approach that is less sensitive to noise and applicable to other IRNDT approaches in order to improve the defect detection is always interesting. As indicated from the literature, low-rank matrices from RPCA have less noise, and in this study, we study the use of this matrix on different IRNDT approaches.

The following section introduces the methods and materials regarding this study.

## 3. Methods and Materials

### 3.1. Robust Principal Component Analysis (RPCA)

The Robust PCA problem can be solved via convex optimization that minimizes a combination of the nuclear norm and the $\ell^{1}$-norm. The augmented Lagrange multiplier (ALM) is a method to solve this convex program. Equation (1) introduces the general method of ALM for solving constrained optimization problems [29]:

(1) $$\begin{array}{c} \min f(X),\;subject\;to\;\;h(X)=0 \end{array}$$

where $f:R^{n}\to R$ and $h:R^{n}\to R^{m}$. Candès et al. [30] used a convex optimization; the formulation they have used is known as PCP. The observation matrix *D* is assumed to be a combination of the low-rank (*A*) and sparse matrix (*E*):

(2) $$\begin{array}{c} D=A+E \end{array}$$

To minimize the energy function, $\ell_{0}$-norm is used.

(3) $$\begin{array}{c} \min_{A,E}\;rank\left(A\right)+\lambda {∥E∥}_{0}\\ subject\;to\;\;D-A-E=0 \end{array}$$

where λ is a positive and arbitrary balanced parameter to determine the contribution of **A** and **E** in minimizing the objective function. Since Equation (3) is an NP-hard problem, i.e., at least as hard as the hardest problems in non-deterministic polynomial (NP) time, Candès et al. [30] reformulated this equation into a similar convex optimization problem as follows:

(4) $$\begin{array}{c} X=\left(A,E\right)\;\;,\min_{A,E}\left({∥A∥}_{*}+\lambda {∥E∥}_{1}\right)\\ subject\;to\;\;D-A-E=0 \end{array}$$

where ${∥A∥}_{*}$, ${∥E∥}_{1}$ are the nuclear norm of A and $l_{1}$-norm of E, respectively. The balance parameter λ is defined as:

(5) $$\lambda =1/\sqrt{\max(m,n)}$$

The low-rank minimization due to the correlation between the frames provides a framework for background modelling. Lin et al. [31] solved Equation (4) using a generic ALM method. The Lagrange function can be defined as:

(6) $$L\left(X,Y,\mu \right)=f\left(X\right)+\langle Y,h\left(X\right)\rangle +\frac{\mu }{2}{∥h\left(X\right)∥}_{F}^{2}$$

The Lagrange function of Equation (4) is defined as:

(7) $$L\left(A,E,Y,\mu \right)={∥A∥}_{*}+\lambda {∥E∥}_{1}+\langle Y,D-A-E\rangle +\frac{\mu }{2}{∥D-A-E∥}_{F}^{2}$$

where Y is the Lagrange multiplier and the penalty parameter μ is a positive scalar parameter. The inexact augmented Lagrange multiplier (IALM) method used to solve the RPCA problem is shown in Algorithm 1. $Y_{0}$ has been initialized to $Y_{0}=D/J\left(D\right)$ [32], making the objective function value $\langle Y_{0},D\rangle$ reasonably large. In addition, $J\left(D\right)=max({∥A∥}_{2},\lambda^{-1}{∥Y∥}_{\infty })$, where ${∥.∥}_{\infty }$ is the maximum absolute value of the input matrix.

In Step 1 of Algorithm 1, ρ is the learning rate, and $\mu_{0}$ is the initialization of the penalty parameter that influences the convergence speed. In [31], it is proven that the objective function of the RPCA problem (Equation (4)), which is non-smooth, has an excellent convergence property. In addition, it has been proven that to converge to an optimal solution $\left(A^{*},E^{*}\right)$ of the RPCA problem, it is necessary for $\mu_{k}$ to be non-decreasing and $\sum_{k=1}^{+\infty }\mu_{k}^{-1}=+\infty$. The proposed algorithm steps are detailed in the following table.
**Algorithm 1:RPCA via IALM method** 
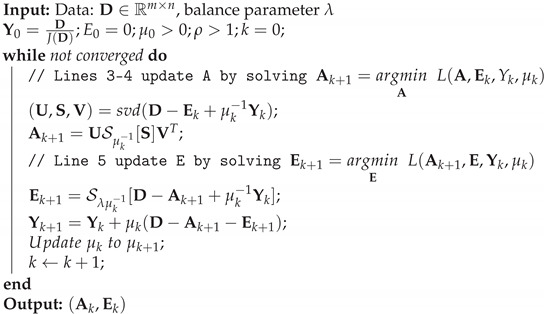



### 3.2. State-of-the-Art

Pulsed thermography has been extensively investigated as a mean to detect defects for a wide variety of applications. Several processing techniques have been proposed and have been thoroughly reported. References [33,34,35] provide a detailed review of various methods. Principal component thermography (PCT) [27], pulsed phase thermography (PPT) [9] and the partial least squares thermography (PLST) [11] are among the most effective.

In this paper, a computed low-rank matrix was used prior to or after the application of PCT, PPT and PLST in the PT regime for comparative purposes.

#### 3.2.1. PCT

PCT was introduced by Rajic et al. [6,27] based on the popular multivariate statistical method, principal component analysis (PCA) [36]. This method constructs a set of empirical orthogonal functions (EOFs), which are strong representations of complex input signals. In IRNDT, PCT tends to project data in the orthogonal space that maximizes the variance of projected data. The EOFs will represent the most critical variability of the data, respectively. In general, the given sequence can be represented with a few EOFs. Typically, the thermal sequence of thousands of frames can be replaced by a maximum of ten EOFs.

#### 3.2.2. PPT

Pulsed phase thermography was introduced by Maldague et al. [9]. Each pixel in the thermal data sequence can be transformed using the one-dimensional discrete Fourier transform (DFT) to extract amplitude and phase information from PT data. Unlike raw thermal data, phase transform ϕ is less sensitive to environmental reflections, emissivity variations, non-uniform heating, surface geometry and orientation. The most important characteristic of this method is that it can provide qualitative and quantitative analysis. For instance, a straightforward formulation of depth estimation (*z*) using the thermal diffusion length μ and the blind frequency $f_{b}$ is:

(8) $$z=C_{1}.\mu =C_{1}.\sqrt{\frac{\alpha }{\pi .f_{b}}}$$

where $f_{b}$ is the frequency at which a given defect has enough contrast to be detected, while $C_{1}$ is the empirical constant and calculated after a series of experiments. It has been observed that $C_{1}\approx 1$ for amplitude data and a value in the range of 1.5 to 2, with $C_{1}=1.82$, are typically adopted for research similar to that presented in [37]. Therefore, probing deeper defects using the phase makes it more interesting than the amplitude. More information regarding PPT can be found in [9].

#### 3.2.3. PLST

PLST [12] is based on a statistical correlation method known as partial least squares regression (PLSR). PLST decomposes predictor X(n×N) and predicted Y(n×M) matrices into loading (*P* and *Q*), score (*T* and *U*) vectors and residuals (*E* and *F*). The predictor matrix corresponds to the thermal profile, while *Y* is defined by the observation time during which the thermal sequence was acquired. Mathematically, the PLS model is expressed as:

(9) $$X=TP^{T}+E$$

(10) $$Y=UQ^{T}+F$$

In order to select the appropriate number of PLS components, two parameters, i.e., the root mean square error (RMSE) and the percentage variance explained in the *X* matrix, must be taken into consideration.

### 3.3. Data Acquisition

**Figure 1:**
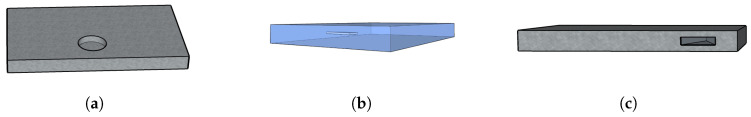
Schematic of a defects in the form of (**a**) flat bottom hole; (**b**) Teflon insert; and (**c**) pullouts.

The experiments were carried out on an academic carbon-fibre-reinforced polymer (CFRP) plate (30.8 cm × 46 cm × 2.57 mm) with 73 defects of 3 different types, i.e., 23 round flat-bottom holes (FBH), 25 triangular Teflon inserts, and pullouts. In order to manufacture the pullout defect, a metallic sheet is removed after polymer curing. Therefore, the pullout can only be located at the edge of the part (Figure 1c). The Teflon insert is made of Teflon sheets inserted between plies (Figure 1b). In the case of FBH manufacturing, a hole is drilled to have a flat reflecting surface at the hole bottom at the backside of the sample (Figure 1a). One of the important defects in non-destructive inspection is delamination, which occurs between plies during manufacturing or by fatigue, bearing damage, impact, etc., during the life-cycle. The academic plate used in this study was prepared to investigate the differences in the thermal response of different artificial defect types. Strictly speaking, all artificial defects are at best an approximation of a real delamination. A pull-out seems to be closer to a real delamination (thermally speaking) but is difficult to produce anywhere other than on the borders of the specimen (which implies that the sample must have an open border). Teflon inserts are traditionally employed for other NDT techniques (e.g., ultrasounds) in thermography. However, Teflon behaves significantly different than a real delamination (air) does. Lastly, flat-bottom-holes are easier to produce, though they are open on the rear side of the specimen and possess a much larger volume than a real delamination. The surface of the specimen possesses a fairly good emissivity, so environmental reflections were negligible. Non-uniform heating had a greater impact on all techniques, as can be seen in Section 4.

**Figure 2:**
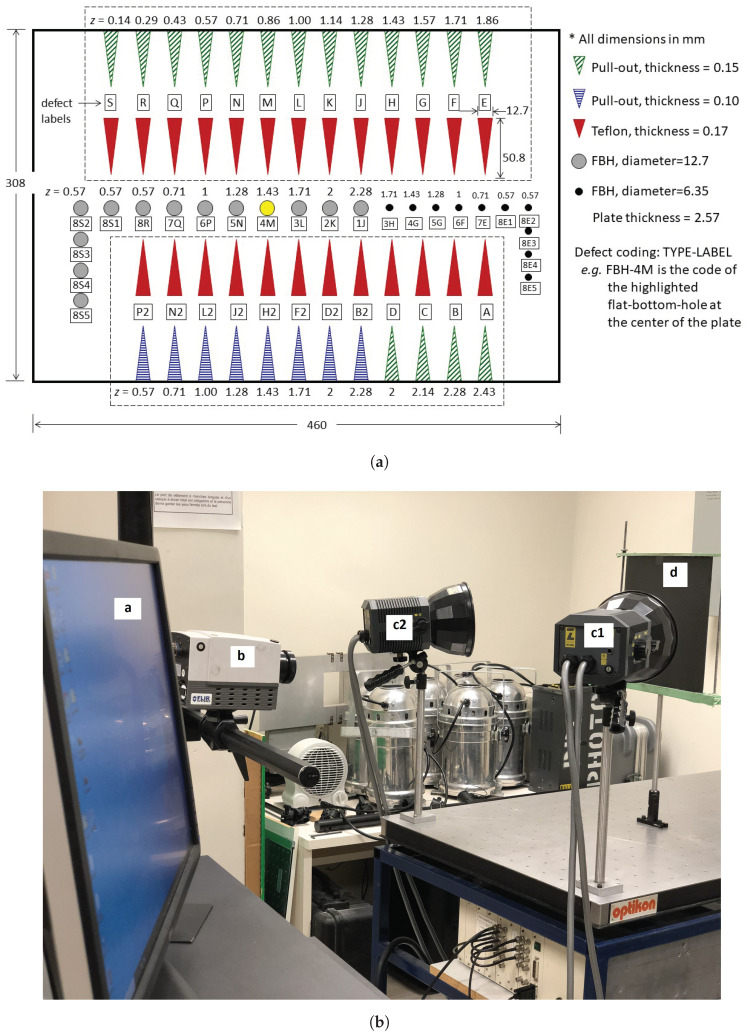
(**a**) CTA CFRP plate, where Z is the defect depth, and labels are used to identify the location of each defect; (**b**) pulsed thermography setup. a, PC; b, IR camera; c1 and c2, left and right flashes; d, CFRP specimen.

The defects vary in size, depth and thickness and are presented in Table 1, and the schematic of the plate shows their respective locations in Figure 2a. The thermophysical properties of CFRP involved in the NDE are: *k*—thermal conductivity (W/m/K), ρ—density (kg/m${}^{3}$) and *c*—specific heat capacity (J/kg/K). The other important thermal properties are: α=k/ρ/c—thermal diffusivity and $e=\sqrt{k\rho c}$—thermal effusivity. The thermophysical information of the CFRP plate is shown in Table 2. The PT experimental setup, two flash lamps for 5 ms sent a thermal pulse (6.4KJ/flash (Balcar, France)) to the specimen; a cooled infrared camera (FLIR Phoenix (FLIR Systems, Inc., Wilsonville, Oregon, USA), InSb, midwave, 3–5 mm, Stirling Cooling) with a frame rate of 180 Hz was used to record the temperature profile in the reflection mode (Figure 2b). The technical camera specifications of the thermal camera are presented in Table 3. The data processing was performed on a PC with 56 GB memory and an Intel(R) Core(TM) i7-4820K control processing unit. Infrared images were taken from a distance of 70 cm by the IR camera without pan nor tilt in a controlled environment.

### 3.4. Metrics

In this section, we added two metrics—one to yield a thermal score indicating thermal anomalies, another to measure the segmentation potential.

#### 3.4.1. Contrast-to-Noise Ratio (CNR)

The signal-to-noise ratio (SNR) is a metric that quantitatively assesses the desired signal quality by estimating the signal level with respect to the background noise. The contrast-to-noise ratio (CNR) is similar to SNR, but it measures the image quality based on the contrast between a defective area and its neighbourhood. Usamentiaga [38] proposed a definition of SNR, which is more robust against noise and image enhancement operations. Equation (11) shows this definition, which has been used in this study. For this purpose, two areas are considered: an area in the defect area (carea) and a region around the defect region as a reference region (narea).

(11) $$CNR=\frac{|\mu_{carea}-\mu_{narea}|}{\sqrt{\frac{\left({\sigma_{carea}}^{2}+{\sigma_{narea}}^{2}\right)}{2}}}$$

where $\mu_{carea}$ and $\mu_{narea}$ are the average levels of contrast in carea and narea, respectively; $\sigma_{carea}$ and $\sigma_{narea}$ are the standard deviation of the contrast in carea and narea, respectively.

#### 3.4.2. Jaccard Similarity Coefficient Score

**Figure 3:**
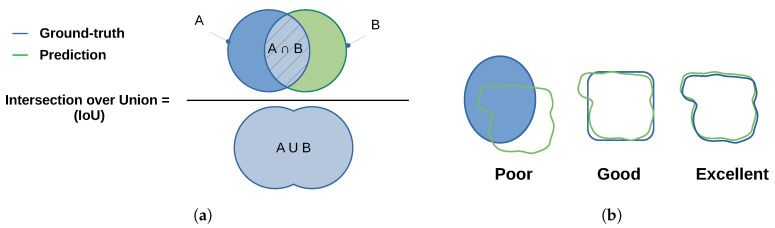
(**a**) Jaccard index similarity definition; (**b**) similarity between the ground-truth and the detected area.

The Jaccard similarity coefficient [39] (also known as Jaccard index or Intersection-Over-Union (IoU)) is a statistical method that emphasizes the similarity between two finite datasets (as illustrated in Figure 3):

This approach mathematically represents Equation (12) and is formally defined as the number of the shared members/pixels between two sets (intersection), divided by the total number of members in either set (union) and multiplied by 100. J(A,B) provides a value between 0 (no similarity) and 1 (identical sets). Hence, the higher the value of IoU, the higher the level of similarities between the two sets (Figure 3b).

(12) $$\begin{array}{c} J\left(A,B\right)=\frac{\left|A\cap B\right|}{\left|A\cup B\right|}=\frac{\left|A\cap B\right|}{\left|A|+|B|-|A\cap B\right|}\;\\ 0\leq J(A,B)\leq 1 \end{array}$$

For the remainder of this article, we will refer to the low-rank matrix A as low-rank matrix (LRM).

### 3.5. Analysis

**Figure 4:**
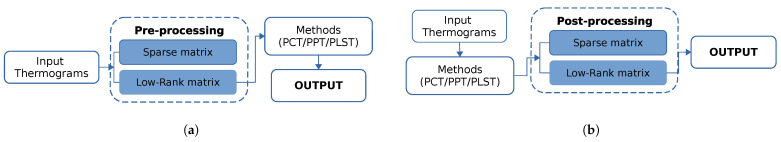
(**a**) Using the method for pre-processing; (**b**) Using the method for post-processing.

The previous section recalls the RPCA we used in our experiments. As described in Figure 4a,b, we conducted two experiments. The main difference between our experiments is that: in the first experiment (Figure 4a), the LRM is computed directly from the raw data; while in the second (Figure 4b), the LRM is computed from the output of the processing methods. For the remainder of this article, we refer to the first experiment as a pre-processing experiment and to the second as a post-processing experiment.

We chose to compare our approach with three state-of-the-art approaches, principal component thermography (PCT) [6,27], pulsed phase thermography (PPT) [9] and partial least-squares thermography (PLST) [11,12], due to the popularity and simplicity of these methods.

**Figure 5:**
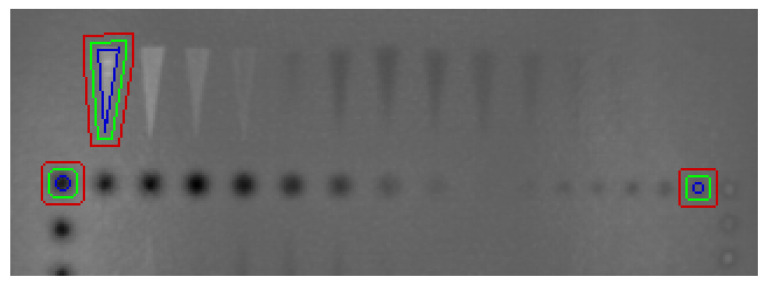
Examples of reference and defect regions. The boundaries of the reference region are between the green and red lines, whilst the defective region is inside the blue line area.

The metrics are computed using different protocols. The defective areas were labelled using LabelMe ^©^ [40]. From the border of the defective region, *n* pixels are considered as a transient region, and from the boundaries of this area, *n* pixels are automatically counted as a non-defective or sound area. Figure 5 illustrates the aforementioned regions so as to estimate the CNR score. According to Equation (11) and the labelled regions, the average and standard deviation values are obtained for all data.

**Figure 6:**
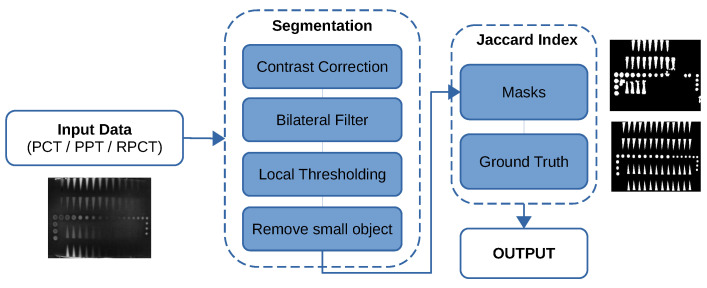
Segmentation and Jaccard index computation flow graph.

Regarding the second metric, Figure 6 depicts the automatic segmentation approach and Jaccard index calculation. In our segmentation approach, after the image’s contrast correction, a bilateral filter [41] smoothed the image. Then, after applying local thresholding, the small artifacts are removed from the image. The obtained mask from the segmentation step can be compared with the ground truth in order to compute the metric score.

## 4. Results

**Figure 7:**
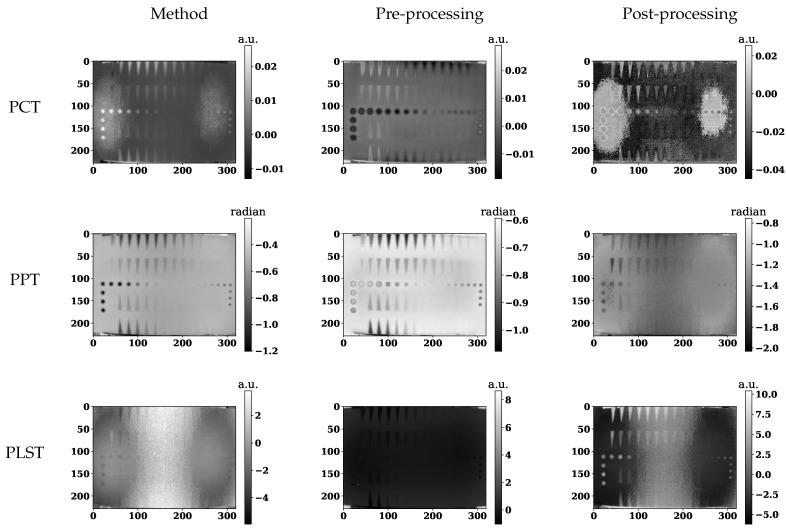
(*1st row*) These images present the 3rd component of PCT data on raw data after using a low-rank matrix for pre-processing and post-processing, respectively. (*2nd row*) These images present PPT data at 0.135 Hz on raw data after using a low-rank matrix for pre-processing and post-processing, respectively. (*3rd row*) These images present the 3rd component of PLST data on raw data after using a low-rank matrix for pre-processing and post-processing, respectively.

The original data acquired by pulsed thermography (raw data) is used as pre- and post-processing for different processing approaches. Figure 7 shows some representative results (selected arbitrarily) of the different methods. The first column in Figure 7 results from different techniques on raw data, where the second column presents RPCA results as a pre-processing method, and the last column shows the RPCA approach used as a post-processing method.

**Figure 8:**
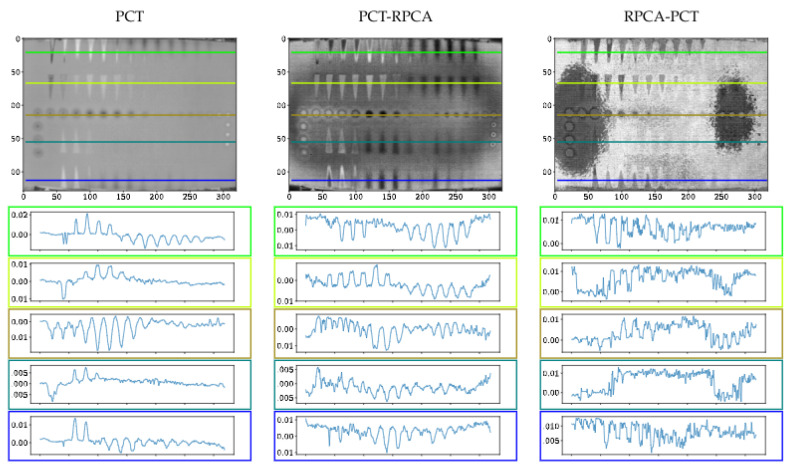
Profiles across the sample after using different processing techniques.

**Figure 9:**
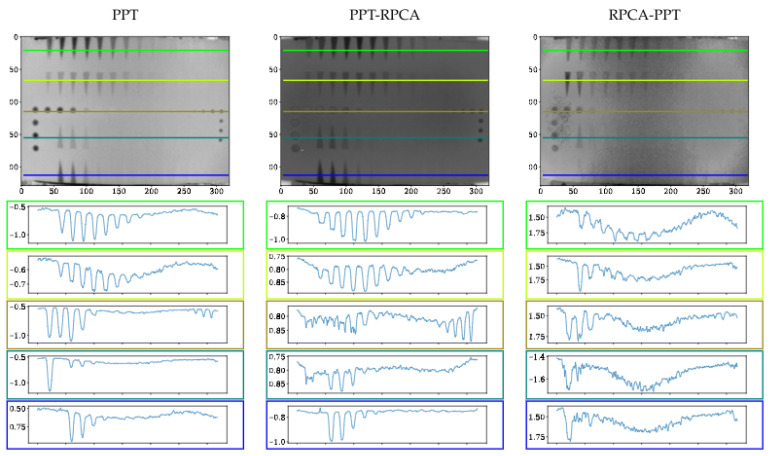
Profiles across the sample after using different processing techniques.

**Figure 10:**
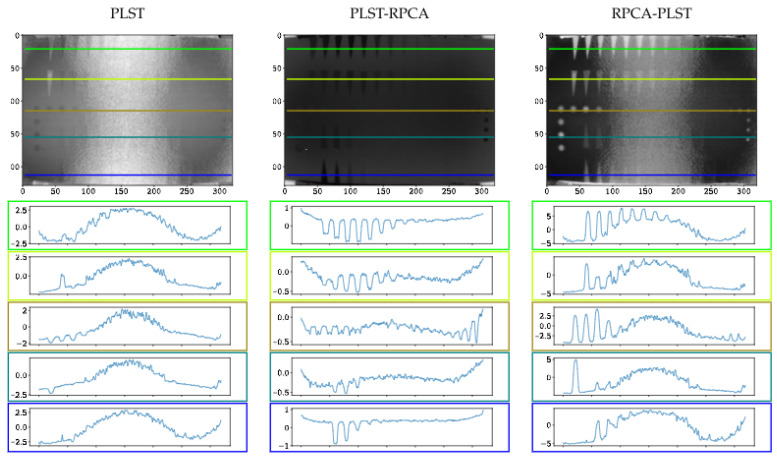
Profiles across the sample after using different processing techniques.

Figure 8, Figure 9 and Figure 10 present the thermal profile across the different lines in images where the defects are either detectable or non-detectable. The first and last lines in each image (green and blue) show the pullout defects profile, while the second and fourth lines (lime and teal) represent the FBHs, and the third line (olive) presents the Teflon inserts profile.

**Figure 11:**
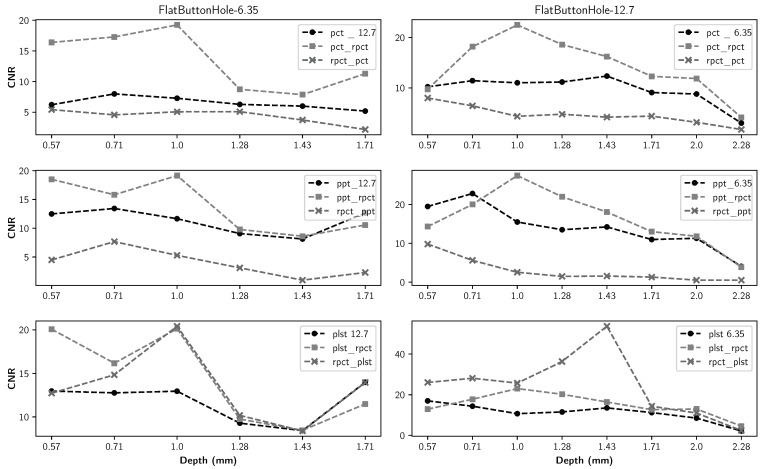
Maximum CNR by different FBHs as a function of defect depth for all data sequences.

**Figure 12:**
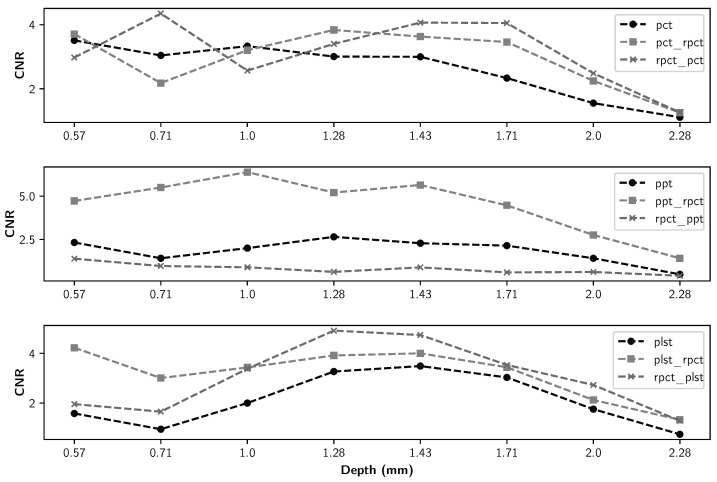
Maximum CNR for pullout-10 as a function of defect depth for all data sequences.

**Figure 13:**
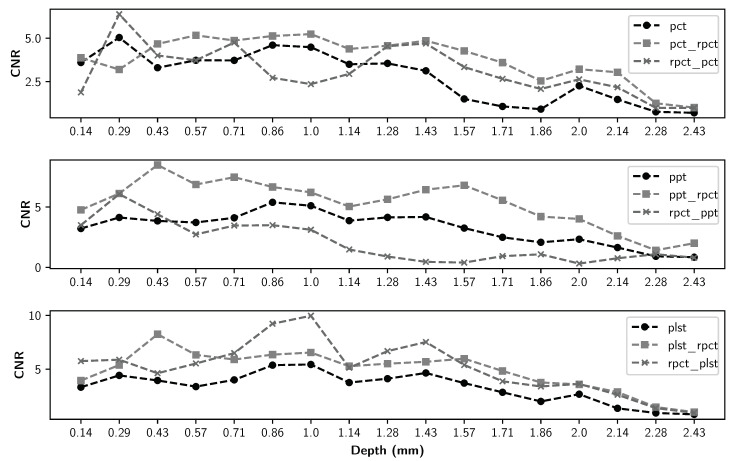
Maximum CNR for pullout-15 as a function of defect depth for all data sequences.

**Figure 14:**
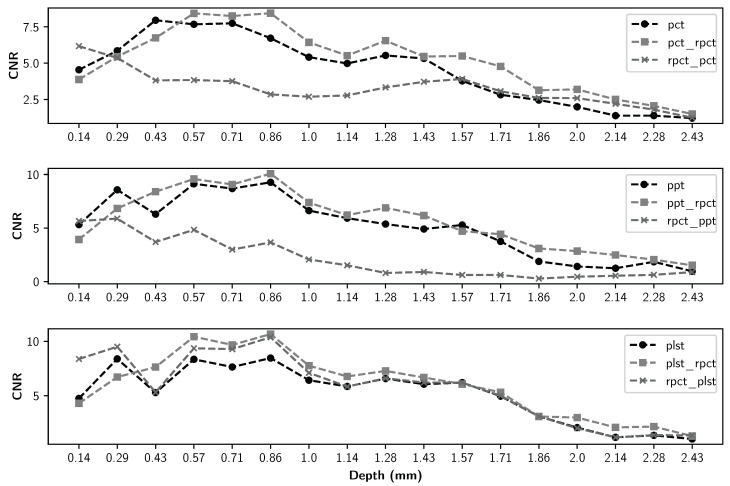
Maximum CNR for teflon insert as a function of defect depth for all data sequences.

The detailed maximum CNR values of all methods for all defect types are presented in Table 4, Table 5 and Table 6. The maximum CNR values between different methods are in bold. Figure 11, Figure 12, Figure 13 and Figure 14 present the maximum CNR value in full sequences for different methods. The CNR values of all defects and all processing techniques were calculated using the defects and reference areas, such as the ones shown in Figure 5.

**Figure 15:**
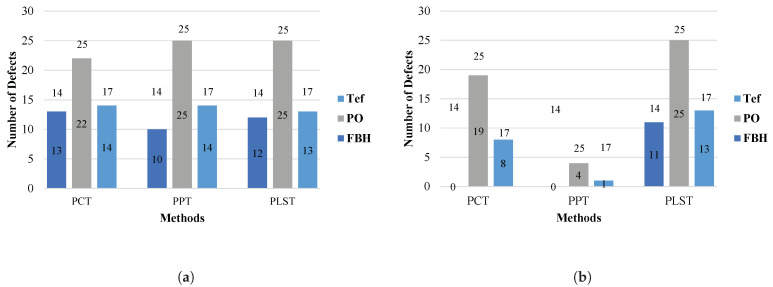
Number of defects that are enhanced for each experiment. (**a**) Results of the pre-processing experiments. (**b**) Results of the post-processing experiments.

Figure 15a,b illustrate the numbers of enhanced defects using pre- and post-processing, respectively. The numbers inside the columns represent the enhanced defects when using different techniques, and the number above the columns are the total number of defects in each case.

The best Jaccard index for all data sequences for different methods is shown in Table 7.

Figure 7 illustrates selected results from different methods. In this figure, the first image from each row presents the selected technique on raw data (PCT, PPT or PLST); the second and third images show the effect of using the LRM as a pre- and post-processing method.

Our segmentation approach was evaluated by the Jaccard index presented in Table 7.

## 5. Discussion

Figure 7 implies that although pre-processing can reduce the non-uniform heating impact, post-processing accentuates this effect. Thermal profiles of different methods across the different lines are shown in Figure 8, Figure 9 and Figure 10. As depicted in the graphs, the flat thermal profiles show the non-defective or sound area, and when the amplitude is increased or decreased, the available discontinuities can be guaranteed. The application of pre-processing before PCT and PPT approaches improved the defect detection; also, in the case of PLST, both pre- and post-processing can increase the detection of anomalies. In addition, the graphs show similar results with quantitative metrics, which will be explained later. From Table 4, Table 5 and Table 6 and Figure 11, Figure 12, Figure 13 and Figure 14, one can note that the results from the pre-processing experiments are noticeably better than those obtained from the post-processing experiment. Note that these results are compared with results obtained without using low-rank matrices for both experiments. For the PCT method, one can note:

- The pre-processing experiments have led to a clear improvement of the results, regardless of the defect type. For 13 of the 14 FBH defects, one can observe an increase in the CNR score. The ratio of this improvement varies from 31.24% to 163.56%. The CNR scores obtained for the PO defects show a higher score in 22 of the 25 defects, with a ratio that varies from 0.43% to 115.88%. Similarly, the CNR scores obtained for the Teflon inserts also show a CNR score increase for 14 of the 17 defects. The ratio of this improvement varies from 2.5% to 80.36%.
- The results of the post-processing experiments do not show any improvement for the FBH defects. Nevertheless, for the PO defects, one can note that there is a higher CNR score for 19 of 25 defects. The ratio of this improvement varies from 0.05% to 149.62%. For Teflon defects, 8 of the 17 defects have a higher CNR score, with a ratio between 2.39% and 58.63%.

From the PPT method results, one can observe:

- As already observed with the PCT, the results of the pre-processing experiments offer an improvement for every type of defect. For 10 of the 14 FBH defects, one can observe that their CNR score increases, with a ratio between 4.58% and 77.19%. The PO defects show an increase in the CNR score for all of the defects. The ratio of improvement varies from 21.72% to 288.97%. For Teflon inserts, the number of defects with a higher CNR is similar to what was observed for the previous method, with 14 of the 17 defects with an improved CNR value. The ratio of improvement varies from 4.43% to 101.45%.
- The results obtained for the post-processing experiment show very little improvement. No improvement at all was recorded for the FBH. For the PO defects, 4 of the 25 defects had an increased CNR value, with a ratio between 8.67% and 46.97%. Only one Teflon defect of the 17 defects had its CNR increased by a ratio of 6.41%.

Finally, from the PLST method results, one can note:

- The pre-processing experiments shows a similar trend as the trend observed for the two other methods. For 12 of the 14 FBH defects, the CNR score increased, with a ratio from 0.43% to 115.88%. All of the PO defects have their CNR score increased, with a ratio between 13.48% and 216.63%. Finally, for the Teflon insert, 13 defects of the 17 obtained an increased CNR score, with a ratio between 7.16% and 77.64%.
- For the post-processing approach, one can note that the results are quite similar to those obtained during the pre-processing experiments. For 11 of the 17 FBH defects, an increase in the CNR value was observed, with a ratio from 9.62% to 296.9%. All of the PO defects show an improvement of their CNR score, ranging from 16.98% to 92.6%. For 13 of the 17 Teflon defects, the CNR score has improved, with a ratio from 0.46% to 76.38%.

Moreover, as indicated in Figure 11, Figure 12, Figure 13 and Figure 14, regarding the relative depths, in all cases (FBHs, POs and TEFs), the deeper the defect, the lower the CNR value (as expected). Comparing the two experiments, one can observe that the pre-processing experiment leads to a larger number of defective regions for PCT and PPT methods than the post-processing experiments. Nevertheless, this observation is not valid for the PLST method, where the results are pretty similar in both experiments. For the PO defect, the increase in terms of CNR score is higher in the pre-processing experiments; the mean ratio of improvement is 2.6 times higher than it is for the post-processing experiments. Similarly, the mean ratio of improvement for the Teflon defects is 1.7 times higher in the pre-processing experiment than in the post-processing experiments. Nonetheless, the mean improvement ratio is 2.5 times higher in the post-processing experiment than in the pre-processing experiment. To conclude, our results show that computing an LRM from the raw data before applying any state-of-the-art method significantly improves the results of the method. In the particular case of FBH defects, one can consider computing an LRM before and after the method.

As one can note in Table 7 and see in Figure 15b, using the LRM, prior to the state-of-the-art processing method, leads to better Jaccard index scores and therefore segmentation in all cases. One can also note that the Jaccard index score for the PLST method does not change much between the pre-processing and post-processing experiments. The Jaccard index score for the PCT and PPT methods decreases noticeably for the segmentation of the post-processing experiment results compared with the segmentation of the raw data. This indicates that the results of the segmentation worsen.

## 6. Conclusions

The present study investigates the benefits of the low-rank matrices for pulsed thermography. The investigation conducted for this study focuses on enhancing defective regions located within a reference sample of CFRP. The sample we used had three types of defects. Two experiments were conducted: during the first experiment, the low-rank matrix was computed from the raw data before applying any processing. During the second experiment, the low-rank matrix is computed from the output of a method, after it was applied on raw data. For both experiments, we used PPT [9], PCT [6,27] and PLST [11,12]. Two figures of merit, the contrast-to-noise ratio (CNR) and the Jaccard similarity coefficient, were used to evaluate the results quantitatively.

Our results conclude that using a low-rank matrix, when used as a pre-processing method, noticeably improves the results of all of the techniques. The low-rank matrix reconstruction effectively reduces the noise and non-uniform heating. When used as a post-processing method, the results vary from one method to another. The results indicate that pre-processing can improve 67.12% of PCT results more than post-processing, especially regarding FBHs (the detectability of FBHs, pullouts and Teflon inserts was increased to 92.86%, 88% and 82.35%, respectively). Furthermore, pre-processing has a better effect on PPT results (67.12% of the defects were detected) than post-processing. For FBHs, pullouts and Teflon inserts, the detectability of defects reached 71.43%, 100% and 82.35%. The detectability of pullouts and Teflon insert defects in both pre- and post-processing has improved, reaching 100% and 76.47%, respectively; however, the detectability is better after using pre-processing in the PLST method. In addition, when used on the output of PLST, the low-rank matrix reconstruction still shows better results than the PLST alone. Nonetheless, this conclusion is not shared for both PPT and PCT. The Jaccard index proved that pre-processing can improve the segmentation potential in all aforementioned methods. In the case of PLST, improvements were made for both pre-processing and post-processing.

This study presents very promising results regarding the improvement of anomaly detection in pulsed thermography in CFRPs. To make the proposed approach more practical in NDT techniques, future research will be directed towards the application of pre- and post-processing on a wider range of materials.

## Figures and Tables

**Table 1:** Defect specifications for the CFRP Plate, Z is the depth of the defect below the inspected surface. Thickness is the defect thickness or thickness of the holes in case of the FBH type of defect.

Defect Code	Z (mm)	Dimensions (mm)	Thickness (mm)	Defect Code	Z (mm)	Dimensions (mm)	Thickness (mm)	Defect Code	Z (mm)	Dimensions (mm)	Thickness (mm)
**Teflon Inserts**				**Pull-Outs**				**FlatBottom Holes**			
Tef-A	2.43	12.7 × 50.8	0.17	PO15-A	2.43	12.7 × 50.8	0.15	FBH-1J	2.28	12.70	0.29
Tef-B	2.28	12.7 × 50.8	0.17	PO15-B	2.28	12.7 × 50.8	0.15	FBH-2K	2.00	12.70	0.57
Tef-C	2.14	12.7 × 50.8	0.17	PO15-C	2.14	12.7 × 50.8	0.15	FBH-3L	1.71	12.70	0.86
Tef-D	2.00	12.7 × 50.8	0.17	PO15-D	2.00	12.7 × 50.8	0.15	FBH-4M	1.43	12.70	1.14
Tef-E	1.86	12.7 × 50.8	0.17	PO15-E	1.86	12.7 × 50.8	0.15	FBH-5N	1.28	12.70	1.29
Tef-F	1.71	12.7 × 50.8	0.17	PO15-F	1.71	12.7 × 50.8	0.15	FBH-6P	1.00	12.70	1.57
Tef-G	1.57	12.7 × 50.8	0.17	PO15-G	1.57	12.7 × 50.8	0.15	FBH-7Q	0.71	12.70	1.86
Tef-H	1.43	12.7 × 50.8	0.17	PO15-H	1.43	12.7 × 50.8	0.15	FBH-8R	0.57	12.70	2.00
Tef-J	1.28	12.7 × 50.8	0.17	PO15-J	1.28	12.7 × 50.8	0.15	FBH-8S1	0.57	12.70	2.00
Tef-K	1.14	12.7 × 50.8	0.17	PO15-K	1.14	12.7 × 50.8	0.15	FBH-8S2	0.57	12.70	2.00
Tef-L	1.00	12.7 × 50.8	0.17	PO15-L	1.00	12.7 × 50.8	0.15	FBH-8S3	0.57	12.70	2.00
Tef-M	0.86	12.7 × 50.8	0.17	PO15-M	0.86	12.7 × 50.8	0.15	FBH-8S4	0.57	12.70	2.00
Tef-N	0.71	12.7 × 50.8	0.17	PO15-N	0.71	12.7 × 50.8	0.15	FBH-8S5	0.57	12.70	2.00
Tef-P	0.57	12.7 × 50.8	0.17	PO15-P	0.57	12.7 × 50.8	0.15	FBH-3H	1.71	6.35	0.86
Tef-Q	0.43	12.7 × 50.8	0.17	PO15-Q	0.43	12.7 × 50.8	0.15	FBH-4G	1.43	6.35	1.14
Tef-R	0.29	12.7 × 50.8	0.17	PO15-R	0.29	12.7 × 50.8	0.15	FBH-5G	1.28	6.35	1.29
Tef-S	0.14	12.7 × 50.8	0.17	PO15-S	0.14	12.7 × 50.8	0.15	FBH-6F	1.00	6.35	1.57
Tef-B2	2.28	12.7 × 50.8	0.17	PO10-B2	2.28	12.7 × 50.8	0.10	FBH-7E	0.71	6.35	1.86
Tef-D2	2.00	12.7 × 50.8	0.17	PO10-D2	2.00	12.7 × 50.8	0.10	FBH-8E1	0.57	6.35	2.00
Tef-F2	1.71	12.7 × 50.8	0.17	PO10-F2	1.71	12.7 × 50.8	0.10	FBH-8E2	0.57	6.35	2.00
Tef-H2	1.43	12.7 × 50.8	0.17	PO10-H2	1.43	12.7 × 50.8	0.10	FBH-8E3	0.57	6.35	2.00
Tef-J2	1.28	12.7 × 50.8	0.17	PO10-J2	1.28	12.7 × 50.8	0.10	FBH-8E4	0.57	6.35	2.00
Tef-L2	1.00	12.7 × 50.8	0.17	PO10-L2	1.00	12.7 × 50.8	0.10	FBH-8E5	0.57	6.35	2.00
Tef-N2	0.71	12.7 × 50.8	0.17	PO10-N2	0.71	12.7 × 50.8	0.10				
Tef-P2	0.57	12.7 × 50.8	0.17	PO10-P2	0.57	12.7 × 50.8	0.10				

**Table 2:** Thermal properties of the CFRP.

Material	Density $\rho \;({\mathrm{kg}/m}^{3})$	Specific Heat $c\;({J/\mathrm{kg}}^{\circ }K)$	Conductivity $k\;({W/(m}^{\circ }K))$	Diffusivity $\alpha \;(m^{2}/s\;10^{-7})$	Effisivity $e\;(W\;s^{0.5}/\left(m^{2}{\;}^{\circ }K\right))$
CFRP (⊥)	1600	1200	0.8	4.167	1239.3

**Table 3:** Technical specification of Phoenix Thermal Camera from FLIR Systems.

Thermal Camera Specifications	
**Parameters**	**Values**
Detector	Indium Antimonide (InSb)
Spectral Range	1.5–5.0 microns
Cold Filter Bandpass	3.0–5.0 μm standard
Resolution	320 × 256 pixels
Detector size	30 × 30 µm
Well Capacity	18 M electrons
Integration Type	Snapshot
Integration Time (Electronic shutter speed)	9 µs to full frame time
Sensor Assembly f/#	f/2.5 standard, f/4.1 optional
Sensor Cooling	Stirling closed cycle cooler; optional Liquid Nitrogen (LN2)
Lens Mount	Bayonet Twist-Lock
Spec Performance (Thermal resolution)	<25 milliKelvin
Dynamic Range	14 bits
Max Frame Rates with RTIE Electronics	320 × 256: 120 frames per sec in full frame; 13.6 kHZ in smallest window (2 × 64)
Max Frame Rates with DAS Electronics	320 × 256: 345 frames per sec in full frame; 38 kHZ in smallest window (2 × 128)

**Table 4:** Maximum CNR values for all data regarding Flat bottom holes in different depths and diameters.

			PCT					PLST					PPT				
**Defect**	**Z**	**Dim.**	**On** **Raw data**	**Pre-P.**	**Post-P.**	**Pre-P vs.** **PCT**	**Post-P vs.** **PCT**	**On** **Raw Data**	**Pre-P.**	**Post-P.**	**Pre-P. vs.** **LST**	**Post-P. vs.** **PLST**	**On ** **Raw data**	**Pre-P.**	**Post-P.**	**Pre-P. vs.** **PPT**	**Post-P. vs. ** **PPT**
**FBH-8E1**	0.57	6.35	6.22	**16.39**	5.42	163.56%	–12.87%	12.97	**20.07**	12.72	54.71%	–1.91%	12.48	**18.50**	4.48	48.18%	–64.08%
**FBH-7E**	0.71	6.35	7.98	**17.28**	4.57	116.38%	–42.82%	12.76	**16.16**	**14.84**	26.61%	16.27%	13.43	**15.82**	7.67	17.77%	–42.86%
**FBH-6F**	1	6.35	7.28	**19.24**	5.07	164.38%	–30.36%	12.96	**20.13**	**20.41**	55.36%	57.54%	11.65	**19.15**	5.29	64.32%	–54.6%
**FBH-5G**	1.28	6.35	6.28	**8.73**	5.08	39.14%	–18.99%	9.28	**9.75**	**10.17**	5.07%	9.62%	9.07	**9.79**	3.11	7.88%	–65.7%
**FBH-4G**	1.43	6.35	6	**7.86**	3.73	31.24%	–37.84%	8.42	**8.45**	8.38	0.43%	–0.49%	8.13	**8.61**	0.98	5.82%	–87.99%
**FBH-3H**	1.71	6.35	5.18	**11.28**	2.18	117.75%	–57.93%	13.99	11.49	13.95	–17.87%	–0.24%	12.65	10.56	2.30	–16.52%	–81.78%
**FBH-8R**	0.57	12.7	10.22	9.74	8	–4.67%	–21.74%	16.99	12.91	**26.08**	–24.06%	53.45%	19.49	14.31	9.81	–26.56%	–49.67%
**FBH-7Q**	0.71	12.7	11.43	**18.17**	6.44	58.91%	–43.64%	14.36	**17.80**	**28.17**	24%	96.18%	22.82	20.03	5.6	–12.25%	–75.47%
**FBH-6P**	1	12.7	11.01	**22.48**	4.37	104.14%	–60.29%	10.68	**23.06**	**25.76**	115.88%	141.17%	15.49	**27.44**	2.54	77.19%	–83.59%
**FBH-5N**	1.28	12.7	11.17	**18.59**	4.78	66.38%	–57.21%	11.53	**20.25**	**36.35**	75.59%	215.16%	13.5	**21.99**	1.48	62.89%	–89.05%
**FBH-4M**	1.43	12.7	12.35	**16.22**	4.21	31.35%	–65.92%	13.53	**16.44**	**53.71**	21.49%	296.9%	14.22	**18.05**	1.57	26.97%	–88.99%
**FBH-3L**	1.71	12.7	9.08	**12.29**	4.40	35.39%	–51.5%	11.22	**12.67**	**14.37**	12.86%	28.04%	10.97	**13.01**	1.31	18.51%	–88.1%
**FBH-2K**	2	12.7	8.79	**11.86**	3.18	34.85%	–63.88%	8.50	**12.99**	**10.99**	52.79%	29.22%	11.3	**11.82**	0.52	4.58%	–95.41%
**FBH-1J**	2.28	12.7	3.02	**4.14**	1.76	37.02%	–41.88%	2.15	**4.56**	**2.39**	112.19%	11.12%	4.06	3.87	0.51	-4.83%	–87.4%

**Table 5:** Maximum CNR values for all data regarding Teflon inserts in different depths and diameters.

			PCT					PLST					PPT				
**Defect**	**Z**	**Dim.**	**On ** **Raw data**	**Pre-P.**	**Post-P.**	**Pre-P vs. ** **PCT**	**Post-P vs. ** **PCT**	**On** **Raw data**	**Pre-P.**	**Post-P.**	**Pre-P. vs.** **PLST**	**Post-P. vs.** **PLST**	**On ** **Raw data**	**Pre-P.**	**Post-P.**	**Pre-P. vs.** **PPT**	**Post-P. vs. ** **PPT**
**Tef-S**	0.14	12.7 × 50.8	4.54	3.88	**6.17**	−14.66%	35.82%	4.75	4.3	**8.38**	−9.45%	76.38%	5.32	3.93	**5.66**	−26.07%	6.41%
**Tef-R**	0.29	12.7 × 50.8	5.85	5.45	5.36	−6.87%	−8.39%	8.39	6.72	**9.51**	−19.92%	13.33%	8.57	6.83	5.88	−20.29%	−31.39%
**Tef-Q**	0.43	12.7 × 50.8	7.95	6.74	3.81	−15.28%	−52.04%	5.29	**7.64**	**5.31**	44.45%	0.51%	6.29	**8.4**	3.7	33.43%	−41.24%
**Tef-P**	0.57	12.7 × 50.8	7.67	**8.43**	3.84	9.83%	−50.01%	8.35	**10.44**	**9.36**	24.97%	12.14%	9.13	**9.58**	4.84	4.89%	−47.02%
**Tef-N**	0.71	12.7 × 50.8	7.74	**8.24**	3.76	6.46%	−51.48%	7.64	**9.67**	**9.29**	26.58%	21.54%	8.69	**9.07**	2.99	4.43%	−65.56%
**Tef-M**	0.86	12.7 × 50.8	6.72	**8.44**	2.85	25.58%	−57.58%	8.47	**10.67**	**10.38**	26.07%	22.56%	9.27	**10.08**	3.66	8.72%	−60.55%
**Tef-L**	1	12.7 × 50.8	5.41	**6.43**	2.69	18.86%	−50.32%	6.43	**7.77**	**7.11**	20.78%	10.52%	6.63	**7.38**	2.07	11.26%	−68.83%
**Tef-K**	1.14	12.7 × 50.8	4.98	**5.52**	2.78	10.87%	−44.27%	5.85	**6.78**	5.85	15.75%	−0.15%	5.92	**6.2**	1.52	4.76%	−74.28%
**Tef-J**	1.28	12.7 × 50.8	5.53	**6.55**	3.33	18.4%	−39.83%	6.58	**7.28**	**6.61**	10.72%	0.46%	5.37	**6.89**	0.81	28.21%	-84.99%
**Tef-H**	1.43	12.7 × 50.8	5.32	**5.46**	3.72	2.5%	−30.19%	6.07	**6.68**	**6.22**	10.05%	2.47%	4.91	**6.17**	0.9	25.65%	−81.63%
**Tef-G**	1.57	12.7 × 50.8	3.78	**5.49**	**3.91**	45.37%	3.44%	6.21	6.07	6.21	−2.21%	−0.03%	5.28	4.71	0.62	−10.9%	−88.29%
**Tef-F**	1.71	12.7 × 50.8	2.82	**4.78**	**3.06**	69.26%	8.54%	4.96	**5.32**	**5**	7.16%	0.75%	3.75	**4.42**	0.62	17.85%	−83.54%
**Tef-E**	1.86	12.7 × 50.8	2.46	**3.13**	**2.6**	27.47%	5.98%	3.1	3.1	3.1	−0.26%	−0.1%	1.88	**3.09**	0.28	64.31%	−84.92%
**Tef-D**	2	12.7 × 50.8	1.99	**3.19**	**2.59**	60.61%	30.41%	2.07	**2.99**	2.01	44.54%	−2.95%	1.41	**2.85**	0.46	101.45%	−67.82%
**Tef-C**	2.14	12.7 × 50.8	1.39	**2.5**	**2.2**	80.36%	58.63%	1.18	**2.09**	1.18	77.64%	0%	1.25	**2.49**	0.55	99.52%	−56.02%
**Tef-B**	2.28	12.7 × 50.8	1.39	**2.06**	**1.8**	48.47%	29.82%	1.36	**2.16**	**1.38**	59.35%	1.88%	1.85	**2.05**	0.63	10.86%	-65.83%
**Tef-A**	2.43	12.7 × 50.8	1.21	**1.5**	**1.24**	23.56%	2.39%	1.02	**1.29**	**1.35**	26.42%	32%	0.97	**1.52**	0.88	57.56%	−8.49%

**Table 6:** Maximum CNR values for all data regarding Pullouts in different depths and diameters.

			PCT					PLST					PPT				
**Defect**	**Z**	**Dim.**	**On ** **Raw data**	**Pre-P.**	**Post-P.**	**Pre-P vs. ** **PCT**	**Post-P vs. ** **PCT**	**On** **Raw data**	**Pre-P.**	**Post-P.**	**Pre-P. vs.** **PLST**	**Post-P. vs.** **PLST**	**On ** **Raw data**	**Pre-P.**	**Post-P.**	**Pre-P. vs.** **PPT**	**Post-P. vs. ** **PPT**
**PO10-P2**	0.57	12.7 × 50.8	3.51	**3.71**	2.98	5.7%	−15.21%	1.58	**4.23**	**1.96**	167.02%	23.75%	2.33	**4.73**	1.391	103.23%	−40.17%
**PO10-N2**	0.71	12.7 × 50.8	3.04	2.18	**4.35**	−28.38%	42.98%	0.95	**3.01**	**1.66**	216.63%	74.21%	1.41	**5.5**	0.966	288.97%	−31.68%
**PO10-L2**	1	12.7 × 50.8	3.33	3.21	2.57	−3.75%	−22.88%	2	**3.44**	**3.38**	71.58%	68.78%	2	**6.39**	0.892	219.71%	−55.38%
**PO10-J2**	1.28	12.7 × 50.8	3.01	**3.84**	**3.4**	27.65%	13.18%	3.27	**3.92**	**4.92**	19.79%	50.34%	2.65	**5.21**	0.628	96.57%	−76.3%
**PO10-H2**	1.43	12.7 × 50.8	3	**3.63**	**4.07**	21.05%	35.62%	3.49	**4**	**4.74**	14.67%	35.79%	2.28	**5.65**	0.887	147.24%	−61.16%
**PO10-F2**	1.71	12.7 × 50.8	2.33	**3.46**	**4.05**	48.24%	73.61%	3.03	**3.44**	**3.54**	13.48%	16.58%	2.14	**4.47**	0.598	108.63%	−72.1%
**PO10-D2**	2	12.7 × 50.8	1.55	**2.25**	**2.48**	44.72%	60.05%	1.76	**2.13**	**2.73**	21.01%	55.41%	1.41	**2.76**	0.623	95.19%	−55.94%
**PO10-B2**	2.28	12.7 × 50.8	1.11	**1.26**	**1.26**	13.05%	13.59%	0.75	**1.33**	**1.29**	78.79%	73.15%	0.49	**1.42**	0.403	189.57%	−17.59%
**PO15-S**	0.14	12.7 × 50.8	3.6	**3.87**	1.87	7.62%	−47.93%	3.33	**3.94**	**5.75**	18.48%	72.78%	3.22	**4.76**	**3.498**	47.84%	8.67%
**PO15-R**	0.29	12.7 × 50.8	5.04	3.19	**6.38**	−36.62%	26.69%	4.42	**5.39**	**5.88**	21.79%	32.96%	4.13	**6.12**	**6.073**	48.21%	46.97%
**PO15-Q**	0.43	12.7 × 50.8	3.3	**4.67**	**4**	41.54%	21.42%	3.95	**8.25**	**4.62**	108.57%	16.94%	3.85	**8.49**	**4.402**	120.52%	14.37%
**PO15-P**	0.57	12.7 × 50.8	3.72	**5.16**	**3.72**	38.69%	0.05%	3.38	**6.33**	**5.53**	87.11%	63.41%	3.72	**6.87**	2.728	84.62%	−26.67%
**PO15-N**	0.71	12.7 × 50.8	3.72	**4.86**	**4.75**	30.8%	27.68%	4.01	**5.9**	**6.49**	47.19%	61.94%	4.11	**7.47**	3.462	82.03%	−15.68%
**PO15-M**	0.86	12.7 × 50.8	4.6	**5.12**	2.72	11.44%	−40.94%	5.38	**6.35**	**9.22**	18.2%	71.53%	5.39	**6.66**	3.494	23.54%	−35.19%
**PO15-L**	1	12.7 × 50.8	4.48	**5.23**	2.35	16.83%	−47.53%	5.44	**6.55**	**9.96**	20.38%	83.21%	5.11	**6.22**	3.117	21.72%	−38.97%
**PO15-K**	1.14	12.7 × 50.8	3.49	**4.38**	2.94	25.31%	−15.83%	3.76	**5.28**	**5.14**	40.36%	36.63%	3.87	**5.04**	1.476	30.23%	−61.86%
**PO15-J**	1.28	12.7 × 50.8	3.55	**4.56**	**4.54**	28.72%	27.93%	4.12	**5.51**	**6.68**	33.88%	62.17%	4.14	**5.64**	0.895	36.18%	−78.38%
**PO15-H**	1.43	12.7 × 50.8	3.12	**4.85**	**4.69**	55.61%	50.38%	4.65	**5.69**	**7.52**	22.38%	61.81%	4.18	**6.44**	0.453	54.13%	−89.15%
**PO15-G**	1.57	12.7 × 50.8	1.5	**4.26**	**3.33**	184.84%	122.65%	3.71	**5.98**	**5.39**	61.21%	45.45%	3.25	**6.8**	0.392	109.1%	−87.95%
**PO15-F**	1.71	12.7 × 50.8	1.06	**3.59**	**2.66**	237.22%	149.62%	2.86	**4.83**	**3.88**	69.32%	35.73%	2.49	**5.56**	0.925	123.3%	−62.84%
**PO15-E**	1.86	12.7 × 50.8	0.91	**2.54**	**2.07**	179.52%	128.19%	2.01	**3.77**	**3.4**	87.79%	69.29%	2.07	**4.21**	1.079	102.84	−47.97
**PO15-D**	2	12.7 × 50.8	2.25	**3.21**	**2.63**	42.41%	16.46%	2.68	**3.59**	**3.63**	34.03%	35.67%	2.33	**4.01**	0.309	72.26	−86.73
**PO15-C**	2.14	12.7 × 50.8	1.47	**3.03**	**2.17**	106.75%	47.78%	1.36	**2.88**	**2.63**	111%	92.6%	1.64	**2.61**	0.752	59.22	−54.09
**PO15-B**	2.28	12.7 × 50.8	0.75	**1.25**	**0.98**	65.25%	30.24%	0.93	**1.48**	**1.38**	58.89%	48.18%	0.91	**1.41**	**1.085**	55.04	18.97
**PO15-A**	2.43	12.7 × 50.8	0.7	**1**	**0.98**	43.19%	41.03%	0.81	**1.01**	**0.95**	25.84%	17.39%	0.84	**2**	0.802	137.81	−4.64

**Table 7:** Jaccard index values for different methods on segmented data.

Method	On Raw Data	Pre_Processing	Post_Processing
**PCT**	60.43	64.08	53.94
**PPT**	61.19	62.82	55
**PLST**	50.66	55.36	55.35

## Data Availability

Data sharing not applicable.

## Abbreviations

The following abbreviations are used in this manuscript:

ASM	Active Shape Model
ALM	Augmented Lagrangian Multiplier
APG	Accelerated Proximal Gradient
a.u	arbitrary units
CFRP	Carbon Fiber Reinforced Plastic
CIS	Cold Image Subtraction
CNN	Convolutional neural network
CNR	Contrast to Noise Ratio
DFT	DiscreteFourier Transform
DRPCA	Double Robust Principal Component Analysis
EALM	Exact Augmented Lagrange Multiplier
ECT	Eddy Current Thermography
ECPT	Eddy Current Pulsed Thermography
EOF	Empirical Orthogonal Functions
ESPCA	Edge-Group Sparse Principal Component Analysis
ESPCT	Edge-Group Sparse Principal Component Thermography
FBH	Flat Bottom Holes
GPGPU	General-purpose computing on graphics processing units
IALM	Inexact Augmented Lagrange Multiplier
ICA	Independent Component Analysis
IoU	Intersection over Union
IRNDT	Infrared Non-Destructive Testing
IRT	InfraRed Thermography
LADMAP	Linearized Alternating Direction Method with Adaptive Penalty
LatLRRT	Latent Low-Rank Representation Thermography
LN	Liquid Nitrogen
LRM	Low-Rank Matrix
MWIR	Mid-Wave InfraRed
NDT	Non Destructive Testing
NMF	Non-negative Matrix Factorization
NP	Non-Deterministic Polynomial
OIALM	Orthogonal Inexact Augmented Lagrange Multiplier
PCA	Principal Component Analysis
PCP	Principal Component Pursuit
PCT	Principal Component Thermography
PLS	Partial Least Square
PLSR	Partial Least Square Regression
PLST	Partial Least Square Thermography
PO	pullouts
PPT	Pulsed Phase Thermography
PT	Pulsed Thermography
RMSE	Root Mean Square Error
ROI	region of interest
RPCA	Robust Principal Component Analysis
RPCT	Robust Principal Component Thermography
SNR	Signal to Noise Ratio
SPCA	Sparse Principal Component Analysis
SPCT	Sparse Principal Component Thermography
SVM	Support Vector Machine
Tef	Teflon Inserts
TSR	Thermographic Signal Reconstruction
TRPCA	Tensor RPCA
UT	Ultrasound Testing
WIALM	Weighted contraction IALM

## References

[1] Vo Dong P.A., Azzaro-Pantel C., Cadene A.L. (2018). Economic and environmental assessment of recovery and disposal pathways for CFRP waste management. Resour. Conserv. Recycl..

[2] Abrate S. (1994). Impact on laminated composite materials. Appl. Mech. Rev..

[3] Fleuret J., Ibarra-Castanedo C., Ebrahimi S., Maldague X. Latent Low Rank Representation Applied to Thermography. Proceedings of the 2020 International Conference on Quantitative InfraRed Thermography.

[4] Khodayar F., Lopez F., Ibarra-Castanedo C., Maldague X. (2017). Optimization of the inspection of large composite materials using robotized line scan thermography. J. Nondestruct. Eval..

[5] Shepard S.M., Rozlosnik A.E., Dinwiddie R.B. (2001). Advances in pulsed thermography. Thermosense XXIII.

[6] Rajic N. (2002). Principal Component thermography for flaw contrast enhancement and flaw depth characterization in composite structures. Compos. Struct..

[7] Wang Q., Hu Q., Qiu J., Pei C., Li X., Zhou H., Xia R., Liu J. (2019). Image enhancement method for laser infrared thermography defect detection in aviation composites. Opt. Eng..

[8] Alard C., Lupton R.H. (1998). A Method for Optimal Image Subtraction. Astrophys. J..

[9] Maldague X., Marinetti S. (1996). Pulse phase infrared thermography. J. Appl. Phys..

[10] Ebrahimi S., Fleuret J., Klein M., Théroux L.D., Georges M., Ibarra-Castanedo C., Maldague X. (2021). Robust Principal Component Thermography for Defect Detection in Composites. Sensors.

[11] Lopez F., Nicolau V., Maldague X., Ibarra-Castanedo C. Multivariate infrared signal processing by partial least-squares thermography. Proceedings of the 16th International Symposium on Applied Electromagnetics and Mechanics.

[12] Lopez F., Ibarra-Castanedo C., de Paulo Nicolau V., Maldague X. (2014). Optimization of pulsed thermography inspection by partial least-squares regression. Ndt Int..

[13] Bouwmans T., Zahzah E.H. (2014). Robust PCA via principal component pursuit: A review for a comparative evaluation in video surveillance. Comput. Vis. Image Underst..

[14] Fan J., Gao Y., Wu Z., Li L. (2017). Infrared Dim Small Target Detection Technology Based on RPCA. DEStech Transactions on Computer Science and Engineering.

[15] Wan M., Gu G., Qian W., Ren K., Chen Q., Zhang H., Maldague X. (2018). Total Variation Regularization Term-Based Low-Rank and Sparse Matrix Representation Model for Infrared Moving Target Tracking. Remote Sens..

[16] Xu Y., Wu Z., Chanussot J., Wei Z. (2018). Joint Reconstruction and Anomaly Detection From Compressive Hyperspectral Images Using Mahalanobis Distance-Regularized Tensor RPCA. IEEE Trans. Geosci. Remote Sens..

[17] Zhu P., Cheng Y., Banerjee P., Tamburrino A., Deng Y. (2019). A novel machine learning model for eddy current testing with uncertainty. Ndt Int..

[18] Draganov I.R., Mironov R.P., Neshov N.N., Manolova A.H. Wild animals population estimation from Thermograph-IC videos using tensor decomposition. Proceedings of the 14th International Conference On Communications, Electromagnetics and Medical Applications 2019 (CEMA’19).

[19] Draganov I., Mironov R. Tracking of Domestic Animals in Thermal Videos by Tensor Decompositions. Proceedings of the New Approaches for Multidimensional Signal Processing: Proceedings of International Workshop, NAMSP 2020.

[20] Liang Y., Bai L., Shao J., Cheng Y. Application of Tensor Decomposition Methods In Eddy Current Pulsed Thermography Sequences Processing. Proceedings of the 2020 International Conference on Sensing, Measurement & Data Analytics in the Era of Artificial Intelligence (ICSMD).

[21] Li G., Zheng Z., Shao Y., Shen J., Zhang Y. Automated Tire Visual Inspection Based on Low Rank Matrix Recovery. https://www.researchgate.net/publication/347083889_Automated_Tire_Visual_Inspection_Based_on_Low_Rank_Matrix_Recovery/fulltext/5fdd1aaf299bf14088228f8a/Automated-Tire-Visual-Inspection-Based-on-Low-Rank-Matrix-Recovery.pdf.

[22] Wu T., Gao B., Woo W.L. (2020). Hierarchical low-rank and sparse tensor micro defects decomposition by electromagnetic thermography imaging system. Philos. Trans. R. Soc..

[23] Cao J., Yang G., Yang X., Li J. A Visual Surface Defect Detection Method Based on Low Rank and Sparse Representation. http://www.ijicic.org/ijicic-160104.pdf.

[24] Wang Q., Paynabar K., Pacella M. (2021). Online automatic anomaly detection for photovoltaic systems using thermography imaging and low rank matrix decomposition. J. Qual. Technol..

[25] Kaur K., Mulaveesala R. Statistical Post-processing Approaches for Active Infrared Thermography: A Comparative Study. Proceedings of the 2021 IEEE 11th Annual Computing and Communication Workshop and Conference (CCWC).

[26] Sun S., Ren H., Dan T., Wei W. (2021). 3D segmentation of lungs with juxta-pleural tumor using the improved active shape model approach. Technol. Health Care.

[27] Rajic N. (2002). Principal Component Thermography.

[28] Hermosilla-Lara S., Joubert P.Y., Placko D., Lepoutre F., Piriou M. Enhancement of open-cracks detection using a principal component analysis/wavelet technique in photothermal nondestructive testing. Proceedings of the 6th International Conference on Quantitative InfraRed Thermography.

[29] Bertsekas D.P. (1982). Enlarging the region of convergence of Newton’s method for constrained optimization. J. Optim. Theory Appl..

[30] Candès E.J., Li X., Ma Y., Wright J. (2011). Robust Principal Component Analysis?. J. ACM.

[31] Lin Z., Chen M., Ma Y. (2010). The augmented lagrange multiplier method for exact recovery of corrupted low-rank matrices. arXiv.

[32] Candès E., Recht B. (2008). Exact Matrix Completion via Convex Optimization. Found. Comput. Math..

[33] Maldague X. (2001). Theory and Practice of Infrared Technology for Nondestructive Testing.

[34] Maldague X.P.V., Moore P.O. (2001). Nondestructive Testing Handbook: Infrared and Thermal Testing.

[35] Ibarra-Castanedo C., Genest M., Piau J.M., Guibert S., Bendada A., Maldague X.P. (2007). Active infrared thermography techniques for the nondestructive testing of materials. Ultrasonic and Advanced Methods for Nondestructive Testing and Material Characterization.

[36] Wold S., Esbensen K., Geladi P. (1987). Principal component analysis. Chemom. Intell. Lab. Syst..

[37] Busse G. (1994). Nondestructive evaluation of polymer materials. Ndt Int..

[38] Usamentiaga R., Ibarra-Castanedo C., Maldague X. (2018). More than Fifty Shades of Grey: Quantitative Characterization of Defects and Interpretation Using SNR and CNR. J. Nondestruct. Eval..

[39] Jaccard P. Lois de Distribution Florale dans la Zone Alpine. Bulletin de la Société vaudoise des sciences naturelles. https://www.e-periodica.ch/digbib/view?pid=bsv-002:1902:38::503#110.

[40] Wada K. labelme: Image Polygonal Annotation with Python. https://github.com/wkentaro/labelme.

[41] Tomasi C., Manduchi R. Bilateral filtering for gray and color images. Proceedings of the Sixth International Conference on Computer Vision (ICCV).

